# Randomised, non-comparative phase II study of weekly docetaxel with cisplatin and 5-fluorouracil or with capecitabine in oesophagogastric cancer: the AGITG ATTAX trial

**DOI:** 10.1038/sj.bjc.6605522

**Published:** 2010-01-12

**Authors:** N C Tebbutt, M M Cummins, T Sourjina, A Strickland, G Van Hazel, V Ganju, D Gibbs, M Stockler, V Gebski, J Zalcberg

**Affiliations:** 1 Department of Medical Oncology, Austin Health, PO Box 5555, Studley Road, Heidelberg, Melbourne, Victoria 3084, Australia; 2 National Health and Medical Research Council Clinical Trials Centre, University of Sydney, Locked Bag 77, Camperdown, Sydney, New South Wales 1450, Australia; 3 Monash Medical Centre, 246 Clayton Road, Clayton, Melbourne, Victoria 3168, Australia; 4 Sir Charles Gairdner Hospital, Hospital Avenue, Nedlands, Perth, Western Australia 6009, Australia; 5 Frankston Hospital, PO Box 52, Frankston, Melbourne, Victoria 3199, Australia; 6 Christchurch Hospital, 2 Riccarton Avenue, Addington, Christchurch, Canterbury 8011, New Zealand; 7 Peter MacCallum Cancer Centre, St Andrews Place, East Melbourne, Melbourne, Victoria 3002, Australia

**Keywords:** advanced gastric cancer, advanced oesophageal cancer, cisplatin, 5-fluorouracil, capecitabine, docetaxel

## Abstract

**Background:**

Docetaxel administered 3-weekly with cisplatin and 5-fluorouracil leads to better survival than does standard therapy in patients with oesophagogastric cancer, but leads to high rates of haematological toxicity. Weekly docetaxel is associated with less haematological toxicity. This randomised phase II study tested weekly docetaxel-based combination chemotherapy regimens, with the aim of maintaining their activity while reducing toxicity.

**Methods:**

Patients with histologically confirmed metastatic oesophageal or gastric carcinoma were randomised to receive weekly docetaxel (30 mg m^−2^) on days 1 and 8, cisplatin (60 mg m^−2^) on day 1, and 5-fluorouracil (200 mg m^−2^ per day) continuously, every 3 weeks (weekly TCF, wTCF); or docetaxel (30 mg m^−2^) on days 1 and 8 and capecitabine (1600 mg m^−2^ per day) on days 1–14, every 3 weeks (weekly TX, wTX).

**Results:**

A total of 106 patients were enrolled (wTCF, *n*=50; wTX, *n*=56). Response rates, the primary end point, were 47% with wTCF and 26% with wTX. Rates of febrile neutropenia were low in each arm. Median progression-free and overall survival times were 5.9 and 11.2 months for wTCF and 4.6 and 10.1 months for wTX, respectively.

**Conclusion:**

Weekly TCF and TX have encouraging activity and less haematological toxicity than TCF administered 3-weekly. Weekly docetaxel-based combination regimens warrant further evaluation in this disease.

Oesophagogastric cancer is a major public health problem and is the fourth highest cause of cancer-related mortality globally (Kamangar *et al*, 2006). Although chemotherapy can improve survival and maintain quality of life for patients with advanced oesophagogastric cancer (Glimelius *et al*, 1995), optimal chemotherapy for this disease has not been defined.

A recent randomised phase III study showed that adding docetaxel to cisplatin and 5-FU (TCF) improved response rates, progression-free survival (PFS) times, and overall survival (OS) (Van Cutsem *et al*, 2006). Although the TCF regimen improved clinical outcomes, it was also associated with toxicity, particularly that related to myelosuppression, with a 29% incidence of febrile neutropenia or neutropenic infection.

Several studies have compared weekly with 3-weekly treatment with docetaxel. Weekly docetaxel is associated with minimal myelosuppression, but with a higher rate of cumulative fatigue, tearing, and nail toxicity (Engels and Verweij, 2005). We postulated that combination regimens using weekly docetaxel may provide palliative benefit for patients with advanced oesophagogastric cancer. As there is potential synergy between docetaxel and capecitabine (Nadella *et al*, 2002), thought to be mediated through activation of thymidine phosphorylase by docetaxel, we also aimed to explore the activity of a doublet regimen, thereby avoiding the potential toxicity of cisplatin. We therefore evaluated two novel regimens based on weekly docetaxel in a randomised phase II design called the ATTAX study (A randomised phase Two study evaluating a weekly schedule of doceTAXel with cisplatin and 5-FU (weekly TCF, wTCF) or with capecitabine (weekly TX, wTX) in advanced oesophagogastric cancer).

## Patients and methods

### Eligibility

The ATTAX study was conducted according to a protocol reviewed and approved by the Australasian Gastro-Intestinal Trials Group (AGITG), and reviewed and approved by the Human Research Ethics Committee of each participating institution. All patients provided written informed consent.

Patients were eligible if they were 18 years of age or older with a histologically confirmed diagnosis of oesophageal, gastric, or oesophagogastric junction carcinoma (squamous, adenocarcinoma or undifferentiated), and of metastatic disease that was unidimensionally measurable according to the Response Evaluation Criteria in Solid Tumours (RECIST). Inclusion of oesophageal, oesophagogastric, and gastric cancers is justified by data showing comparable outcomes for these diseases (Chau *et al*, 2009a). Similarly, outcomes for patients with advanced squamous cell carcinoma are not significantly different from those for patients with adenocarcinoma (Chau *et al*, 2007). Patients were not allowed to have had previous anticancer treatment, except for adjuvant radiotherapy or chemotherapy completed at least 12 months before. Further inclusion criteria included WHO performance status (PS) of 0, 1, or 2 (PS2 patients were required to have serum albumin of at least 30 g l^−1^); adequate bone marrow function, including platelets (>100 × 10^9^ cells per l) and neutrophils (>1.5 × 10^9^ cells per l); normal renal function, including normal serum creatinine and calculated creatinine clearance of at least 50 ml min^−1^; and adequate hepatic function, including serum total bilirubin <1.25 × upper limit of normal range, alanine transaminase or aspartate transaminase <2.5 × upper limit of normal range, and alkaline phosphatase <5 × upper limit of normal range. In addition, patients had to be able to swallow tablets, have a life expectancy of more than 12 weeks, and have no concurrent uncontrolled medical conditions and no previous malignant disease other than non-melanotic skin cancer or carcinoma *in situ* of the uterine cervix or other cancers treated with curative intent at least 5 years previously and without evidence of relapse. Patients were required to have a negative pregnancy test and had to agree to practise adequate contraception.

Exclusion criteria included medical or psychiatric conditions that compromised the patient's ability to give informed consent or comply with the study protocol, metastatic disease of the central nervous system, pregnancy or breastfeeding, clinical evidence of peripheral neuropathy of >grade II, and significant deafness or uncontrolled tinnitus. Patients with any uncontrolled concurrent medical condition, known malabsorption syndrome, or who had participated in an investigational drug study within 4 weeks were also excluded from participation in the study.

### Randomisation, stratification and treatment

The ATTAX study was a randomised, phase II, open-label, multicentre study of wTCF or wTX. Randomisation was carried out centrally at the coordinating centre, and patients were stratified by WHO PS (0, 1 *vs* 2) and institution.

Patients were randomly assigned in equal proportions to receive either docetaxel (Taxotere; Sanofi-Aventis, Paris, France) (30 mg m^−2^) on days 1 and 8, cisplatin (60 mg m^−2^) on day 1, and 5-fluorouracil (200 mg m^−2^ per day) by continuous infusion, every 3 weeks (wTCF); or docetaxel (30 mg m^−2^) on days 1 and 8 and oral capecitabine (Xeloda; Roche, Basel, Switzerland) (800 mg m^−2^) twice daily on days 1–14, every 3 weeks (wTX). The dosages were selected for the combination regimens with reference to earlier phase I or II studies (Chen *et al*, 2002; Lee *et al*, 2006; Mrozek *et al*, 2006). Premedication of dexamethasone (8 mg) was given before docetaxel administration, and cisplatin hydration was given according to each investigator's routine practice. Dose-modification criteria were defined in the protocol. Treatment continued for eight cycles in the absence of disease progression, any request by the patient or physician to discontinue therapy, unacceptable toxicity, pregnancy, or serious systemic allergic reaction to any of the study drugs. At the investigator's discretion, patients with no disease progression or grade III or IV toxicity could continue beyond eight cycles. Patients in the wTCF arm experiencing auditory or peripheral neurotoxicity or renal impairment, thought to be related to cisplatin, were allowed to substitute carboplatin for cisplatin.

### Evaluation and outcomes

Before randomisation, each patient was assessed by complete physical examination, full blood count, clotting profile, blood biochemistry, tumour markers (carcinoembryonic antigen (CEA) and carbohydrate antigen (CA 19.9)), 12-lead electrocardiogram, contrast-enhanced CT scan of the thorax, abdomen, and pelvis, and a pregnancy test for women of child-bearing potential.

Subsequently, complete physical examination, blood biochemistry, and a toxicity and adverse event assessment were repeated before each cycle began; a full blood count was repeated before every docetaxel infusion. A tumour marker assessment and contrast-enhanced CT scan of the thorax, abdomen, and pelvis were repeated at the end of every second treatment cycle, then 12-weekly until disease progression.

Toxicity was graded according to the National Cancer Institute Common Terminology Criteria for Adverse Events (NCI CTCAE), version 3.0.

Quality of life was assessed using the European Organisation for Research and Treatment of Cancer Quality-of-Life Questionnaire (QLQ) C30, version 3.0 (01 February 2003), together with the oesophageal-specific module (OES 18) or the gastric module (STO 22). Patients with tumours involving the oesophagogastric junction completed the oesophageal module. Questionnaires were completed 3-weekly for the first 12 weeks, then 6-weekly until the completion of chemotherapy, then 12-weekly until disease progression.

After permanent discontinuation of study treatment, patients were assessed for progression status (until documented disease progression), commencement of non-study treatment, and survival status every 12 weeks until death.

### Statistical analysis

The primary clinical end point of the study was response rate, as assessed by RECIST. Secondary end points were OS, PFS, treatment-related toxicity, disease-associated symptoms, and quality of life.

Although randomisation was used to allocate patients to either the wTCF or wTX arm, no comparisons between treatment regimens were planned. The purpose of randomisation was to reduce bias due to patient selection into either treatment arm.

Overall survival was measured from the date of randomisation to the date of death from any cause. Progression-free survival was measured from the date of randomisation to the first evidence of disease progression or the date of death if progression was not previously documented. Time-to-event parameters were estimated using the Kaplan–Meier method. Disease-associated symptoms were derived from the QLQs.

The study used Simon's two-stage design in each arm. For each arm, the first stage required more than five confirmed responses (complete or partial) in the first 21 patients. The second stage involved complete accrual to 50 patients per treatment arm.

Each treatment was expected to achieve a response rate of 37%, which was considered clinically worthwhile and consistent with previous studies using docetaxel. The lowest limit of therapeutic effect considered to be of interest was a response rate of 17%. On the basis of these limits, and 90% power and a 95% confidence level, 13 or more responses (complete or partial) per treatment arm were required to determine that a regimen was active.

### Study monitoring

An independent data and safety monitoring board reviewed the safety data after 15 and 25 patients had been enrolled in each treatment arm. The tumour response of each patient was centrally reviewed by the lead study clinician and a clinician independent of the study. A total of 7% of patients at 14% of institutions were audited by an independent auditor. No significant protocol discrepancies or deviations were noted.

## Results

### Patient characteristics

**Figure 1:**
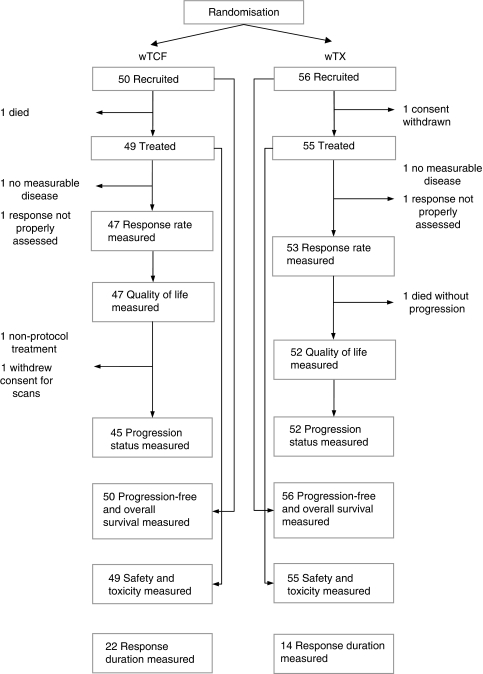
Enrolment and analysis in the ATTAX study. wTCF=weekly docetaxel, plus cisplatin and 5-fluorouracil; wTX=weekly docetaxel plus capecitabine.

Between June 2004 and May 2006, 106 patients were randomised (wTCF, 50 patients; wTX, 56 patients) from 19 institutions in Australia and 1 in New Zealand. Two patients were ineligible (no measurable disease), two patients did not commence treatment (one died and one withdrew consent), and two patients did not have any subsequent valid RECIST tumour assessments (Figure 1). The 100 patients who commenced treatment, who had measurable disease, and were assessed according to RECIST, were included in the response-rate analysis. All 106 patients randomised to the study were included in the PFS and OS analyses on an intention-to-treat basis. The 104 patients who commenced study treatment were included in the toxicity analyses. The 99 patients who completed the QLQs were included in the disease-related symptoms and quality-of-life analyses.

Baseline characteristics were well balanced between the treatment arms (Table 1).

### Treatment

The median number of cycles delivered per patient was 6 cycles of wTCF (range, 1–8) and 5 cycles of wTX (range, 1–14). Dose intensities compared with the starting dosages in the wTCF arm were docetaxel, 92% cisplatin, 91% and 5-FU, 98%. In the wTX arm, they were docetaxel, 98% and capecitabine, 93%. Four patients in the wTCF arm had cisplatin-related toxicity and subsequently substituted carboplatin for cisplatin. Treatment delays of more than 1 week occurred for 10 patients (29%) in the wTCF arm and for 4 patients (7%) in the wTX arm.

### Efficacy

Interim response analysis was carried out after 21 patients were recruited to each treatment arm. There were 11 partial responses in the wTCF arm and 5 partial responses in the wTX arm, meeting the criterion of at least 5 responses per treatment arm for the study to continue.

A total of 106 patients were recruited, and the final response analysis was performed 12 months after the last patient was randomised. Of the 47 patients assessable for response in the wTCF arm, 2 had complete response, 20 had partial response, and 18 had stable disease. Of the 53 assessable patients in the wTX arm, none had complete response, 14 had partial response, and 28 had stable disease (Table 2). The confirmed overall response rates were 47% (95% CI, 32–62%) for wTCF and 26% (95% CI, 15–40%) for wTX.

At the median follow-up time of 40.7 months, 92 patients had progressed, two could not be assessed for progression (one commenced non-protocol treatment before progression and one withdrew consent for further CT scans), whereas four patients in the wTCF arm and one patient in the wTX arm had not progressed. The median durations for response were 6.45 months for wTCF and 6.74 months for wTX.

**Figure 2:**
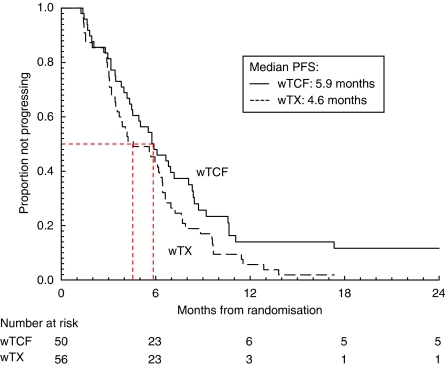
Kaplan–Meier curves of progression-free survival for advanced oesophagogastric cancer patients treated with weekly docetaxel, plus cisplatin and 5-fluorouracil (wTCF; *n*=50), or weekly docetaxel with capecitabine (wTX; *n*=56).

**Figure 3:**
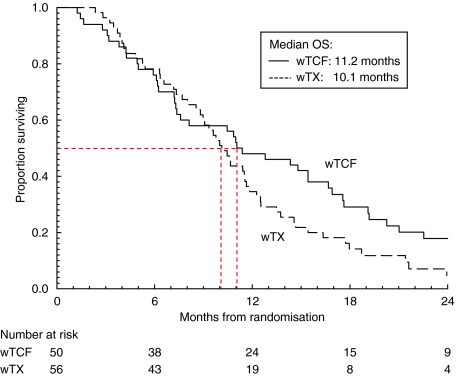
Kaplan–Meier curves of overall survival for advanced oesophagogastric cancer patients treated with weekly docetaxel along with cisplatin and 5-fluorouracil (wTCF; *n*=50), or weekly docetaxel with capecitabine (wTX; *n*=56).

At the time of analysis, 98 patients had died. Median PFS times were 5.9 months for wTCF and 4.6 months for wTX (Figure 2). Median OS times were 11.2 months for wTCF and 10.1 months for wTX (Figure 3).

### Toxicity

Toxicity in the 104 patients who commenced treatment is summarised in Table 3. Three patients on wTCF and one on wTX had grade III febrile neutropenia; both rates are significantly less than the rates observed with 3-weekly docetaxel-based chemotherapy regimens. The most significant common adverse events in the wTCF arm were grade III or IV diarrhoea, grade III or IV fatigue, grade III stomatitis, grade III anorexia, and grade III nausea. The most significant common adverse events in the wTX arm were grade III nausea, grade III vomiting, grade III diarrhoea, grade III anorexia, and grade III fatigue.

Mortality from any cause at 60 days was 6% in the wTCF arm (three patients; 95% CI, 0.7–11.3%) and 0% in the wTX arm (95% CI, 0–6%). There were no treatment-related deaths.

### Disease-associated symptoms and quality of life

Improvement in a specific disease-associated symptom or an aspect of quality of life was defined as an increase of 10 points or more for that questionnaire item for more than 3 weeks. For wTCF and wTX, improvement in global health and quality of life was seen in 30% and 35% of patients, in nausea and vomiting in 30% and 31%, in fatigue in 36% and 46%, and in pain in 47% and 50%, respectively. The most striking improvement was in dysphagia in patients with oesophageal disease, among whom 71% and 70% treated with wTCF and wTX improved, respectively, compared with 46% and 41% of patients with gastric disease, respectively.

## Discussion

This randomised phase II study has shown that it is feasible to develop combination chemotherapy regimens incorporating weekly docetaxel for advanced oesophagogastric cancer. There is clear evidence that docetaxel has non-cross-resistant activity in advanced oesophagogastric cancer. Docetaxel has efficacy as a single agent after failure of platinum and fluoropyrimidines, as well as additive activity when used in combination with cisplatin and 5-FU (Cascinu *et al*, 2001; Van Cutsem *et al*, 2006). However, significant myelosuppression with 3-weekly administration poses a major problem for the development of combination chemotherapy regimens, because the myelosuppressive effects add to the toxicities of other chemotherapy agents. The most striking change in the safety profile of the weekly docetaxel-based combination regimens used in this study was a substantially lower rate of myelosuppression and complicated neutropenia compared with that of 3-weekly TCF (Van Cutsem *et al*, 2006). This occurred despite the somewhat older population of patients (the median age in both arms was over 60 years) in this study, whereas older patients have higher rates of toxicity with 3-weekly TCF (Van Cutsem *et al*, 2006). Cumulative toxicities related to docetaxel, such as fatigue and tearing, did not occur at high frequency in either treatment arm. In addition to modifying the docetaxel schedule, both regimens involved an altered fluoropyrimidine schedule (either continuous-infusion 5FU or capecitabine) compared with 3-weekly TCF, which used a 4-day infusion of high-dose 5FU. This may also have contributed to lower rates of myelosuppression, as other studies have shown more manageable rates of haematological toxicities with protracted infusion schedules of 5FU (Thuss-Patience *et al*, 2005).

We showed clear evidence of efficacy of both regimens, with PFS and OS times that seem promising. Although cross-study comparisons are problematic, the population enrolled did not seem to have more favourable demographic features than those in the study by Van Cutsem *et al*, (2006); hence, an OS of over 10 months in each arm is encouraging. A recent evaluation of over 1000 patients with advanced oesophagogastric cancer identified several prognostic factors affecting survival outcomes (Chau *et al*, 2004) (the Royal Marsden Hospital Prognostic Index), which have been validated in a subsequent data set (Chau *et al*, 2009b). Most patients in each arm in this study had moderate risk factors, previously associated with OS times of 8.6 months, reinforcing the finding that the regimens were clinically active. Palliative benefit was also shown by the improvement in relevant disease-related symptoms, as determined by QLQs.

Several studies in advanced oesophagogastric cancer have shown the activity of docetaxel and capecitabine doublets (Chun *et al*, 2005; Kim *et al*, 2005; Lorenzen *et al*, 2005), although this is not universal (Orditura *et al*, 2006). As a randomised phase II study, our protocol was not powered for direct efficacy comparisons between the two arms. Nevertheless, the wTCF triplet regimen had the most promising clinical efficacy, especially in terms of response rates, and also for PFS and OS. Although the wTCF triplet was tolerable, it is notable that the improved efficacy was associated with higher rates of grade III/IV diarrhoea, stomatitis, fatigue, and febrile neutropenia than the wTX doublet. A similarly designed weekly docetaxel-based triplet was associated with a comparable toxicity profile and encouraging efficacy in a population in which almost half of the patients had locally advanced disease only (Lorenzen *et al*, 2007). Other studies have shown similar trends for greater activity from a triplet regimen than from a doublet regimen in advanced gastric cancer (Ajani *et al*, 2005; Roth *et al*, 2007). In other diseases, such as colorectal cancer or breast cancer, it is common to sequence doublet therapy or even monotherapy regimens. This may not be so effective an approach for advanced oesophagogastric cancer, as patients often have significant tumour-related symptoms at diagnosis, requiring regimens with the highest levels of activity to achieve significant palliative benefit. Furthermore, for those patients with locally advanced disease (without overt metastatic disease), regimens that result in high response rates may achieve tumour downstaging, thereby facilitating subsequent resection (Lorenzen *et al*, 2007).

In recent years, oral fluoropyrimidines have been evaluated in oesophagogastric cancer. Capecitabine has been shown to have equivalent activity to 5-FU, with a different safety profile (Cunningham *et al*, 2008). It is likely that modification of the wTCF regimen by substituting an oral fluoropyrimidine for 5-FU would maintain activity, while potentially improving safety and convenience.

Novel biological targeted agents, such as bevacizumab, cetuximab, and panitumumab, have also improved outcomes in a range of cancers including colorectal, breast, and lung cancer (Hurwitz *et al*, 2004; Sandler *et al*, 2006; Jonker *et al*, 2007; Miller *et al*, 2007; Van Cutsem *et al*, 2007). Some of these agents are currently being evaluated in advanced oesophagogastric cancer. Weekly docetaxel-based chemotherapy provides a useful chemotherapy backbone for evaluation of targeted agents, and the AGITG is currently evaluating the efficacy and safety of adding the epidermal growth factor receptor-targeted antibody, panitumumab, to wTCF chemotherapy as treatment for this disease.

## Figures and Tables

**Table 1:** Patient and cancer baseline characteristics

	**wTCF (*n*=50)**		**wTX (*n*=56)**	
**Characteristic**	**No.**	**%**	**No.**	**%**
*Age (years)*				
Mean (standard deviation)	60.5 (11.5)		59.1 (10.8)	
Range	35–82		32–79	
				
*Gender*				
Male	42	84	42	75
				
*WHO performance status*				
0	21	42	31	55
1	28	56	23	41
2	1	2	2	4
				
*Primary site*				
Oesophagus	11	22	20	36
Oesophagogastric junction	13	26	13	23
Gastric	26	52	23	41
				
*Disease status*				
Local recurrence	2	4	5	9
				
*Histology*				
Squamous cell carcinoma	2	4	9	16
Adenocarcinoma	47	94	45	80
Undifferentiated	1	2	2	4
				
*Sites of metastasis*				
Nodal	30	61	41	73
Liver	25	51	28	50
Pulmonary	7	15	15	27
Peritoneal	10	20	6	11
Bone	5	10	7	13
				
*Number of metastatic organs*				
1	24	48	21	38
2	19	38	24	43
3	3	6	6	11
4	3	6	5	9
				
*RMH prognostic index*				
Good	12	24	19	34
Moderate	37	74	36	64
Poor	1	2	1	2
				
*Received before adjuvant treatment*				
Adjuvant radiotherapy	2	4	1	2

Abbreviations: WHO=World Health Organization; wTCF=weekly docetaxel plus cisplatin and 5-fluorouracil; wTX=weekly docetaxel plus capecitabine.

**Table 2:** Best overall response rates in 100 evaluable patients

	**wTCF (%) (95% CI)**	**wTX (%) (95% CI)**
**Tumour response**	**(*n*=47)**	**(*n*=53)**
Confirmed complete response	4	0
Confirmed partial response	43	26
Confirmed complete or partial response	47 (32–62)	26 (15–40)
Stable disease	38	53
Progressive disease	15	21

Abbreviations: wTCF=weekly docetaxel plus cisplatin and 5-fluorouracil; wTX=weekly docetaxel plus capecitabine.

**Table 3:** Haematological and non-haematological adverse events (NCI CTCAE, version 3.0)

	**wTCF (*n*=49)**		**wTX (*n*=55)**	
**Type of adverse event**	**Grade III, IV or V; No. (%)**	**All grades; No. (%)**	**Grade III, IV or V; No. (%)**	**All grades; No. (%)**
Infection with normal neutrophils	7 (14)	14 (29)	4 (7)	23 (42)
Febrile neutropenia	3 (6)	3 (6)	1 (2)	1 (2)
Anorexia	9 (18)	33 (67)	5 (9)	28 (51)
Nausea	8 (16)	38 (78)	8 (15)	39 (71)
Vomiting	6 (12)	23 (47)	5 (9)	22 (40)
Diarrhoea	11 (22)	30 (61)	5 (9)	27 (49)
Stomatitis	9 (18)	31 (63)	1 (2)	18 (33)
Alopecia	0 (0)	30 (61)	0 (0)	27 (49)
Rash: hand–foot skin reaction or PPE	4 (8)	16 (33)	3 (5)	25 (45)
Allergic reaction or hypersensitivity	0 (0)	1 (2)	0 (0)	2 (4)
Fatigue	9 (18)	45 (92)	5 (9)	47 (85)
Nail changes	0 (0)	12 (24)	2 (4)	27 (49)
Watery eyes (tearing)	0 (0)	12 (24)	0 (0)	18 (33)
Neuropathy: motor	1 (2)	1 (2)	0 (0)	4 (7)
Neuropathy: sensory	0 (0)	14 (29)	1 (2)	16 (29)
Neutrophil count ( × 10^9^ cells per l)	5 (10)	24 (49)	1 (2)	13 (24)
Platelet count ( × 10^9^ cells per l)	0 (0)	1 (2)	0 (0)	1 (2)

Abbreviations: NCI CTCAE=National Cancer Institute Common Terminology Criteria for Adverse Events; wTCF=weekly docetaxel plus cisplatin and 5-fluorouracil; wTX=weekly docetaxel plus capecitabine.
